# Antimicrobial Synergy between Aminoglycosides and Licorice Extract in *Listeria monocytogenes*

**DOI:** 10.3390/pathogens11040440

**Published:** 2022-04-06

**Authors:** Myungseo Park, Liz Horn, Victoria Lappi, Dave Boxrud, Craig Hedberg, Byeonghwa Jeon

**Affiliations:** 1 Division of Environmental Health Sciences, School of Public Health, University of Minnesota, Minneapolis, MN 55455, USA; park2421@umn.edu (M.P.); hedbe005@umn.edu (C.H.); 2 Public Health Laboratory, Minnesota Department of Health, Saint Paul, MN 55164, USA; liz.horn@state.mn.us (L.H.); victoria.lappi@state.mn.us (V.L.); dave.boxrud@state.mn.us (D.B.)

**Keywords:** *Listeria*, antimicrobial synergy, drug potentiation, plant extract, aminoglycosides

## Abstract

*Listeria monocytogenes* is a foodborne pathogen that can develop serious invasive infections. Among foodborne pathogens, *L. monocytogenes* exhibits the highest case fatality despite antibiotic treatment, suggesting the current therapy should be improved. Although ampicillin and gentamicin are used as a combination therapy to treat listeriosis, our results showed there is no synergy between the two antibiotics. We discovered that aqueous extract of licorice generated significant antimicrobial synergy when combined with aminoglycosides, such as gentamicin, in *L. monocytogenes*. In the presence of 1 mg/mL licorice extract, for instance, the minimum inhibitory concentration (MIC) of gentamicin was reduced by 32-fold. Moreover, antimicrobial synergy with licorice extract made gentamicin-resistant clinical isolates of *L. monocytogenes* susceptible to gentamicin. Given the common use of licorice as a food sweetener in Western countries and a herb in Oriental medicine, our findings suggest that licorice extract can be potentially used as an antibiotic adjuvant to improve the efficacy of antimicrobial treatment of listeriosis.

## 1. Introduction

*Listeria monocytogenes* is a foodborne pathogen often isolated from a variety of processed food [1]. Although the incidence of listeriosis is less frequent compared to *Campylobacter* and *Salmonella*, *L. monocytogenes* exhibits the highest case fatality rate among foodborne pathogens; 16.9% in the United States and 18.8% in the EU [2,3]. For antimicrobial therapy for serious cases of listeriosis, aminopenicillin and benzylpenicillin are the drugs of choice [4]. To improve treatment efficacy, penicillin drugs are combined with gentamicin because the combination of the two drugs was reported to make synergy [5]. However, the mortality of listeriosis remains high despite antibiotic treatment [6]. In a retrospective cohort study, combination therapy using penicillin and gentamicin did not decrease the mortality of listeriosis patients compared with penicillin-based monotherapy [7]. This raises the question of whether the combination of these antibiotics can generate antimicrobial synergy or not and suggests novel intervention measures should be developed to improve antimicrobial chemotherapy to treat listeriosis. 

Traditionally, plants are the major source of drug discovery [8,9]. A number of phyto-compounds have been demonstrated to have the capability of reversing antibiotic resistance by interfering with the function of resistance determinants [10]. Especially, a synergy between tea or herb extract with antimicrobials has been reported in Gram-positive and Gram-negative bacteria. The green tea epigallocatechin gallate generates synergy with cefotaxime against extended-spectrum β-lactamase (ESBL)-producing *Escherichia coli* by increasing the generation of reactive oxygen species [11]. *Daphne genkwa* extract potentiated the antibacterial activity of oxacillin against methicillin-resistant *Staphylococcus aureus* (MRSA) [12]. In our previous study, we discovered that some plant-derived antioxidants significantly sensitized *Campylobacter* to clinically-important antibiotics, such as fluoroquinolones and macrolides, by increasing drug permeability and decreasing the production of a multidrug efflux pump [13]. Moreover, a synergy between herbal extracts and drugs has been reported in drug-resistant *Candida*. Recently, Khan, A. *et al*. showed that a combination of methanolic extract of ginger and fluconazole made synergy in the treatment of drug-resistant vulvovaginal candidiasis [14]. Drug potentiators, which are also called antimicrobial adjuvants, are compounds that can enhance the activity of antimicrobials and may complement existing therapy by improving treatment efficacy [15]. Thus far, little has been studied to develop plant-derived drug potentiators to improve antimicrobial therapy for listeriosis.

Most antimicrobial compounds derived from plants, such as polyphenols and essential oils, are hydrophobic; this limits their practical application due to poor solubility in aqueous environments [16]. In addition, the drugs of choice for treating listeriosis (i.e., penicillin and gentamicin) are hydrophilic, suggesting that drug potentiators for combination with these antibiotics should ideally be water-soluble. This is another factor to consider in developing plant-based drug potentiators to improve antimicrobial therapy for listeriosis. In this study, we investigated the capability of water extracts of dietary plants to potentiate antibiotics against *L. monocytogenes*.

## 2. Results

### 2.1. The Combination of Ampicillin and Gentamicin Did Not Generate Antimicrobial Synergy

**Figure 1:**
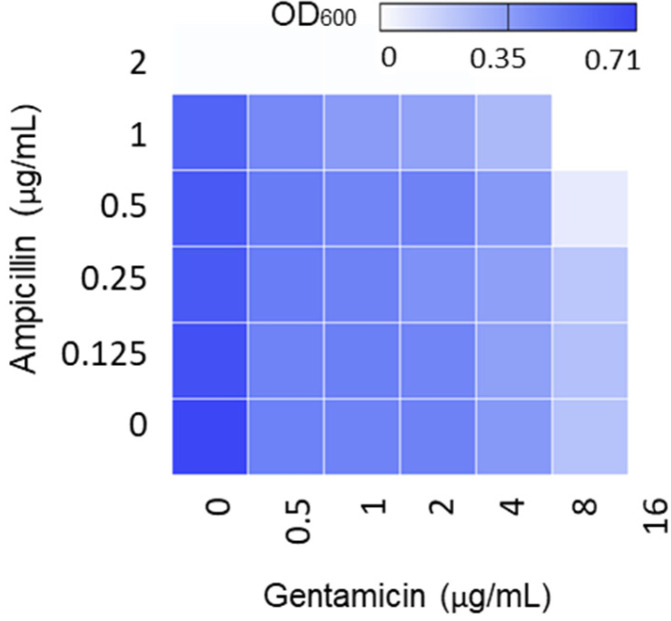
Absence of antimicrobial synergy between ampicillin and gentamicin. The heat map of a checkerboard titration assay shows the growth (OD_600_) of *L. monocytogenes* ATCC 19115 in the presence of gentamicin and ampicillin combinations.

First, we examined if the combination of ampicillin and gentamicin is synergistic because the combination therapy of the two antibiotics is commonly adopted for treating listeriosis but does not appear to be more effective than penicillin-based monotherapy [7]. The synergy between ampicillin and gentamicin was reported based on growth inhibition in an article published more than four decades ago, and information about the fractional inhibitory concentration (FIC) is not available [5]. Thus, we conducted a checkerboard assay to determine the degree of antimicrobial synergy between ampicillin and gentamicin using an FIC index. When ampicillin and gentamicin were combined, the minimum inhibitory concentrations (MICs) of both antibiotics were reduced by 2-fold only at sub-lethal concentrations, such as 1/2 MICs (Figure 1). The FIC indices were ≥1 (data not shown), suggesting the combinations were not synergistic but were deemed as either additive (FIC = 1) or indifferent (FIC > 1) [17]. Although ampicillin and gentamicin are often used as combinatory therapy to treat serious cases of listeriosis, our results suggest these combination does not make antimicrobial synergy. 

### 2.2. Licorice Extract Made Antimicrobial Synergy with Aminoglycosides

Before examining antimicrobial synergy, we evaluated the antimicrobial activity of water extracts from 10 kinds of dietary plants, including baobab, cinnamon, ginger, lemongrass, licorice, moringa, stevia, pomegranate, rosemary, and thyme. Whereas the MIC of licorice extract was 4 mg/mL in *L. monocytogenes*, those of the other plant extracts were ≥16 mg/mL (data not shown). Water extracts of the tested dietary plants other than licorice did not exhibit notable antimicrobial activity in *L. monocytogenes*, although essential oils and ethanol extracts of some of the tested dietary plants, such as rosemary, cinnamon, and thyme, have been reported to be antimicrobial in *L. monocytogenes* in previous studies [18,19,20]. This may be because our study used water extracts; thus, the aqueous extract did not contain hydrophobic anti-listerial compounds. Based on the results, licorice extract was selected for testing antimicrobial synergy in the rest of the study. 

**Figure 2:**
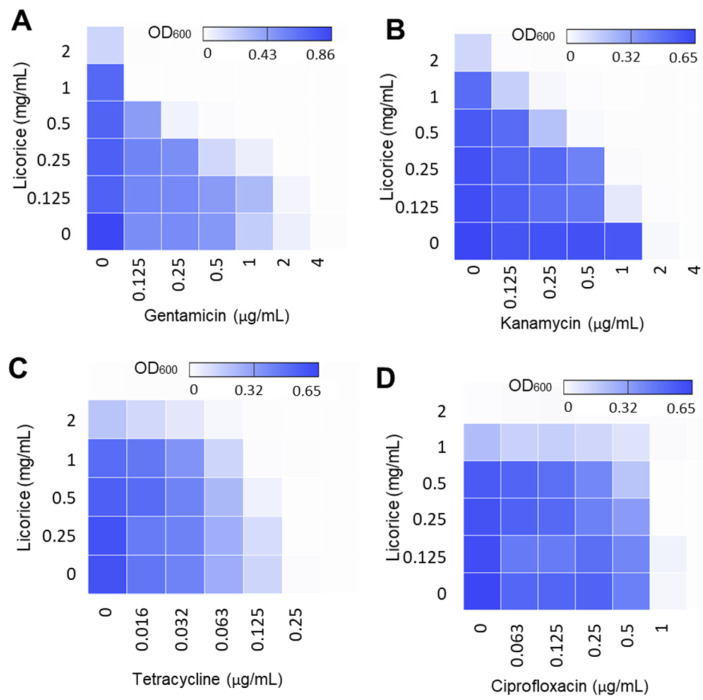
Evaluation of the antimicrobial synergy of licorice extracts in *L. monocytogenes* ATCC 19115 in combination with (**A**) gentamicin, (**B**) kanamycin, (**C**) tetracycline, and (**D**) ciprofloxacin. Heat maps of checkerboard titration assays show the optical density at 600 nm (OD_600_) of *L. monocytogenes*. The results are representative of at least three independent experiments.

To assess the antimicrobial synergy of licorice extract, checkerboard titration assays were performed in combination with different classes of antibiotics (Figure 2). Interestingly, licorice extract generated substantial antimicrobial synergy against *L. monocytogenes* with aminoglycosides (Figure 2A,B). In the presence of 1 mg/mL licorice extract, the MICs of gentamicin and kanamycin were reduced by 32- and 8-fold, respectively (Figure 2A,B). The FIC indices were ≤0.5. when licorice extract was used at ≥0.5 mg/mL (Table 1), suggesting the combinations are synergistic [21].

Since the mode of action of aminoglycosides is the inhibition of protein synthesis, we also examined if licorice extract could make synergy with other antibiotics whose mode of action is protein synthesis inhibition, such as tetracycline, erythromycin, and chloramphenicol. The licorice extract enhanced the antimicrobial activity of tetracycline only when tetracycline was used at 1/2 MIC (Figure 2C) and was not synergistic with erythromycin and chloramphenicol (data not shown). Moreover, the synergy of licorice extract was assessed with ampicillin and ciprofloxacin because ampicillin is the drug of choice for treating listeriosis, and ciprofloxacin is the primary antibiotic for treating gastroenteritis. Licorice extract did not affect the MIC of ampicillin (data not shown) and reduced the MIC of ciprofloxacin by 2-fold when used at ≥0.25 mg/mL (Figure 2D). These results show that licorice extract generates antimicrobial synergy, specifically with aminoglycosides.

### 2.3. Increased Bacteriostatic and Bactericidal Activities of Gentamicin by Licorice Extract

**Figure 3:**
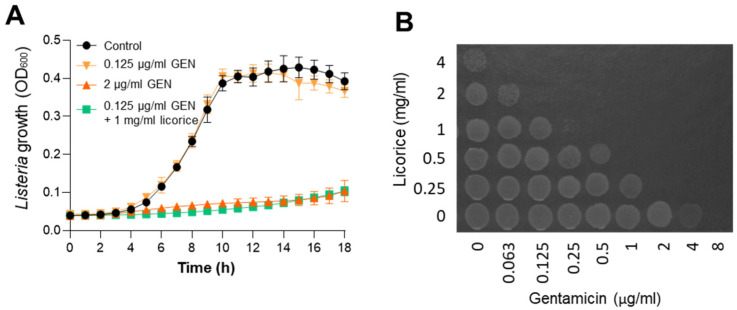
Synergistic growth inhibition and bacterial killing activities of gentamicin and licorice extract combinations against *L. monocytogenes.* (**A**) Synergistic growth inhibition of *L. monocytogenes* by gentamicin and licorice extract combinations. The results show the means and standard deviations of triplicate samples. The experiment was repeated three times and generated similar results. GEN: gentamicin. (**B**) Synergistic bactericidal activity of gentamicin and licorice extract combinations.

The growth of *L. monocytogenes* was monitored in the presence of gentamicin and licorice extract. The supplementation of bacterial cultures with 1 mg/mL of licorice extract enabled gentamicin to completely inhibit the growth of *L. monocytogenes* at 0.125 μg/mL gentamicin, which is 32-fold lower than the MIC of gentamicin (Figure 3A). Moreover, licorice extract markedly increased the bactericidal activity of gentamicin against *L. monocytogenes*; 1 mg/mL of licorice extract decreased minimum bactericidal concentration (MBC) by 16-fold (Figure 3B). Our findings demonstrated that licorice extract synergically enhances both bacteriostatic and bactericidal activities of gentamicin against *L. monocytogenes*. 

### 2.4. Re-Sensitization of Gentamicin-Resistant L. monocytogenes to Gentamicin with Licorice Extract

**Figure 4:**
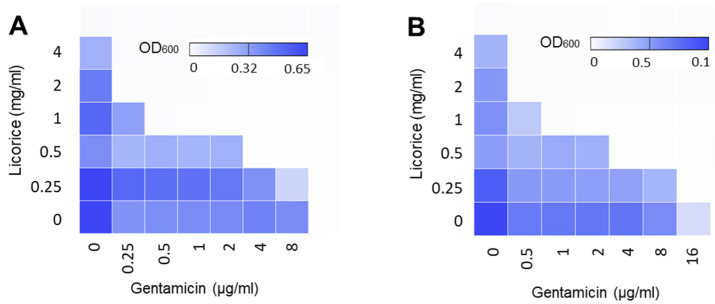
Antimicrobial synergy between gentamicin and aqueous extract of licorice against gentamicin-resistant *L. monocytogenes* strains isolated from clinical cases: (**A**) PNUSAL008195 and (**B**) PNUSAL002708. The heat maps show the optical density at 600 nm (OD_600_) of *L. monocytogenes* in the presence of gentamicin and licorice combinations. The results are representative of at least three independent experiments.

Checkerboard assays were conducted using gentamicin-resistant *L. monocytogenes* strains isolated from human clinical cases to evaluate if antimicrobial synergy with licorice extract can re-sensitize gentamicin-resistant *L. monocytogenes* to gentamicin. Interestingly, licorice extract also made substantial antimicrobial synergy in gentamicin-resistant strains. In the presence of 1 mg/mL licorice aqueous extract, the MIC of gentamicin was reduced by 32-fold in gentamicin-resistant strains (Figure 4A,B). Currently, standard breakpoints to interpret antimicrobial resistance in *L. monocytogenes* are available only for penicillin, carbapenems, macrolides, and trimethoprim-sulfamethoxazole [22], and 1 μg/mL is generally used as the breakpoint of gentamicin in *L. monocytogenes* [23]. Based on the MIC changes by licorice extract, the supplementation of ≥1 mg/mL of licorice extracts completely re-sensitized gentamicin-resistant isolates to gentamicin (Figure 4).

## 3. Discussion

Listeriosis is a rare disease but can develop serious invasive infections, such as bacteremia and meningitis, particularly in people with weak immune systems [24]. For instance, pregnant women are 10~20 times more likely to be infected with *L. monocytogenes* compared to the general population [25]. Adults who are 70 years and older are 4.8 times more likely to get *Listeria* infections in the United States [26]. For treating listeriosis, aminopenicillins, such as ampicillin, are frontline antibiotics [4] and are generally combined with gentamicin to improve treatment efficacy [5]. The prevalence of *L. monocytogenes* resistant to ampicillin or gentamicin varies depending on the geographical location. Studies show that antimicrobial resistance in *L. monocytogenes* is more prevalent in China and Germany compared to other countries. Approximately 4.6% of *L. monocytogenes* strains from food, food-processing plants, and human clinical cases are resistant to gentamicin in Germany [23], and 1.2% of food isolates are gentamicin-resistant in China [27]. In addition to antibiotic resistance, the lack of synergy between ampicillin and gentamicin, as shown in Figure 1, may also explain why combinatory therapy does not outperform penicillin-based monotherapy [7]. 

The high case fatality of listeriosis, such as 16.9% in the United States and 18.8% in the EU [2,3], urgently demands the improvement of antimicrobial therapy for treating listeriosis. For this, however, the characteristics of populations vulnerable to listeriosis should be taken into consideration. Normally, last-resort antibiotics are likely to cause side effects and can be toxic compared to frontline antibiotics [28]. Since pregnant women and older people are the major populations vulnerable to *Listeria* infection, therapies should not incur health burdens and side effects. Moreover, the discretional use of antibiotics in pregnancy is another important factor to consider for the development of antimicrobial therapy to treat listeriosis. For example, fluoroquinolones are a common antibiotic class for treating gastroenteritis, when used empirically [29], but are normally avoided during pregnancy [30]. 

In this study, we improved the activity of gentamicin, a common antibiotic to treat listeriosis, in combination with water extract of licorice; our results demonstrated that licorice extract synergistically enhanced the antimicrobial activity of gentamicin in both gentamicin-sensitive and -resistant *L. monocytogenes*. Licorice has a long history of use in both Eastern and Western countries. Licorice is used in food as a popular sweetening compound [31] and is a common herb used in Oriental medicine and is known to have beneficial health effects, such as antitumor, antiviral, and anti-inflammatory activities [32]. Licorice extract has been reported to have about 122 chemical compounds with various biological activities, including echinatin, isoliquiritigenin, and luteone, which stimulate nuclear factor erythroid 2-related factor 2 (Nrf2) that controls the expression of genes encoding antioxidant enzymes [33]. Glycyrrhizin is a major component of licorice extract and is about 30–50 times sweeter than sucrose [34]. Glycyrrhizin inhibits the infection by influenza virus [35] and may alleviate the severity of Coronavirus Disease 2019 (COVID-19) by interfering with viral entry into the host cells [36,37]. Moreover, licorice extract has antimicrobial effects on bacterial pathogens, such as *S. aureus* [38] and *Streptococcus mutans*, a main oral pathogen causing dental caries [39]. In a clinical trial, mouthwash containing licorice extract inhibited oral pathogens effectively [40]. The findings in this study suggest that licorice extract can possibly be used as a drug potentiator to improve the efficacy of antimicrobial therapy for treating listeriosis.

Interestingly, licorice extracts generated antimicrobial synergy specifically with aminoglycosides, not with antibiotics in other classes. Aminoglycosides are antimicrobial against various Gram-negative bacteria and Gram-positive bacteria, and gentamicin is the most widely used aminoglycoside drug [41]. The mode of action of aminoglycosides involves the inhibition of protein synthesis by binding to the A-site on the 16S ribosomal RNA of 30S ribosome [42]. Although we tested other antibiotic classes inhibiting protein syntheses, such as chloramphenicol and erythromycin, licorice extract did not increase the antimicrobial activity of these antibiotics. At this point, the mechanisms behind the antimicrobial synergy with aminoglycosides are not yet understood. In MRSA, flavonoids from licorice increase membrane permeability and disrupt the proton motive force [43]. In *L. monocytogenes*, we speculate that licorice extract may facilitate the interaction of aminoglycosides with the cellular targets because the synergistic activity is specific to aminoglycosides. Future studies are still needed to elucidate molecular mechanisms underlying the antimicrobial synergy and to identify compounds responsible for the synergy in *L. monocytogenes*. Moreover, *in vivo* studies are required to confirm the synergy between aminoglycosides and licorice for developing therapeutic formulations. Nevertheless, our data suggest that licorice extract has great potential to improve the efficacy of antimicrobial therapy for listeriosis.

## 4. Materials and Methods

### 4.1. Bacterial Strains and Culture

*L. monocytogenes* ATCC 19115 was purchased from the American Type Culture Collection (ATCC). Gentamicin-resistant *L. monocytogenes* strains (PNUSAL008195 and PNUSAL002708) were isolated from invasive listeriosis cases by the Minnesota Department of Health. *L. monocytogenes* strains were aerobically cultured at 37 °C on Brain Heart Infusion (BHI) media.

### 4.2. Antimicrobial Susceptibility Tests

MICs were measured using the microtiter broth dilution method [44] using water extracts from powders from 10 different dietary plants, including baobab (Ecoideas, Newmarket, ON, Canada), cinnamon (McCormick & Company, Inc., Baltimore, MD, USA), ginger (McCormick & Company, Inc), lemongrass (Natrevibe Botanicals, Rahway, NJ, USA), licorice (Banyan Botanicals, Ashland, OR, USA), moringa (Aduna, London, UK), stevia (Natrevibe Botanicals, Rahway, NJ, USA), pomegranate (Natrevibe Botanicals, Rahway, NJ, USA), rosemary (Starwest Botanicals, Sacramento, CA, USA), and thyme (McCormick & Company, Inc.), which were purchased from trusted vendors. Water extraction was conducted as described previously [45] with some modifications to perform extraction at room temperature under light protection without using hot water. The MBC was determined by spotting 5 μL of cultures from the 96-well plate onto BHI agar plates. The plates were incubated at 37 °C aerobically overnight to examine bacterial survival. 

### 4.3. Checkerboard Titration Assay

Checkerboard titration assays were performed as described previously [46]. Briefly, antibiotics and plant extracts were 2-fold serially diluted on each column and row, respectively. Antibiotics were purchased from MilliporeSigma (St. Louis, MO, USA). Suspension of *L. monocytogenes* was added to a 96-well plate (ca., 10^5^ CFU per well), and the plate was incubated at 37 °C for 18 h. After incubation, bacterial growth (OD_600_) was measured with a plate reader (Varioskan, ThermoFisher), and an aliquot (5 μL) was transferred from each well onto BHI agar plates to measure bactericidal activity. The plates were incubated overnight at 37 °C.

### 4.4. Calculation of FIC Index

Synergistic effects were evaluated by determining an FIC index as described elsewhere [17]. The FIC index was calculated as FIC = FIC_A_ + FIC_B_, where FIC_A_ is the MIC of agents A and B in combination divided by the MIC of agent A alone, and FIC_B_ is the MIC of agents A and B in combination divided by the MIC of Agent B alone. An FIC index of ≤0.5 was deemed as synergistic activity [21].

## Figures and Tables

**Table 1:** The FIC index of combinations of licorice extract with antibiotics.

Licorice (mg/mL)	Gentamicin (µg/mL)				Kanamycin (µg/mL)				Tetracycline (µg/mL)				Ciprofloxacin (µg/mL)			
	0.25	0.5	1	2	0.125	0.25	0.5	1	0.016	0.032	0.063	0.125	0.063	0.125	0.25	0.5
0.25	0.63	0.63	0.63	0.53	1.00	0.75	0.63	0.56	2.00	2.00	2.00	2.00	2.00	2.00	2.00	2.00
0.5	**0.19**	**0.19**	**0.19**	**0.09**	0.75	**0.50**	**0.38**	**0.31**	1.00	1.00	1.00	1.00	1.50	1.50	1.50	1.50
1	**0.16**	**0.16**	**0.16**	**0.06**	0.63	**0.38**	**0.25**	**0.19**	0.75	0.75	0.75	0.75	1.50	1.50	1.50	1.50
2	**0.13**	**0.13**	**0.13**	**0.04**	0.53	**0.28**	**0.16**	**0.09**	0.75	0.75	0.75	0.75	1.25	1.25	1.25	1.25
	Synergy (FIC ≤ 0.5)			Additive (0.5 < FIC ≤ 1)					Indifference (1 < FIC < 2)							
